# Proposal for an Expanded Classification of the Superficial Musculoaponeurotic System (SMAS) in the Human Forehead, Based on Anatomical and Microscopic Study

**DOI:** 10.3390/life16050765

**Published:** 2026-05-02

**Authors:** Yuriy L. Vasil’ev, Olesya Kytko, Elena O. Bakhrushina, Irina Smilyk, Pavel Sarygin, Dmitriy Kalinin

**Affiliations:** 1 Department of Operative Surgery and Topographic Anatomy, I.M. Sechenov First Moscow State Medical University, Trubetskaya St., 8, Bld. 2, 119991 Moscow, Russia; kytko_o_v@staff.sechenov.ru; 2 A.P. Nelyubin Institute of Pharmacy, First Moscow State Medical University (Sechenov University), 119435 Moscow, Russia; bakhrushina_e_o@staff.sechenov.ru; 3 Institute of Anatomy LLC, 111024 Skolkovo, Russia; smilyk_i@mail.ru; 4 Federal State Budgetary Institution “A.V. Vishnevsky National Medical Research Center of Surgery” of the Ministry of Health of the Russian Federation, 117997 Moscow, Russia; psarygin@mail.ru (P.S.); dmitry.v.kalinin@gmail.com (D.K.)

**Keywords:** SMAS, forehead anatomy, retaining ligaments, histology, clinical anatomy, arborized smas, fibro-septal smas

## Abstract

Introduction. The superficial musculoaponeurotic system (SMAS) is fundamental for facial soft tissue support and surgical rejuvenation. Although its morphology in the midface and neck is well characterized, the structure of its cranial extension to the forehead remains a subject of terminological uncertainty. The aim of this study was to conduct a detailed histological and immunohistochemical examination of the forehead supporting structures to characterize their morphology and propose an expanded, region-specific classification of the SMAS. Material and methods. Full-thickness tissue specimens (n = 30) were obtained from five standardized facial regions (parotid, buccal, temporal, frontal, and cervical) from 12 male and 18 female body donors (aged 25–70 years). Specimens were processed for histological analysis using hematoxylin and eosin, van Gieson staining, and Masson’s trichrome. Immunohistochemical staining for S100 protein was used to identify neural structures. Morphometric analysis was performed on digitized sections to quantify interseptal distances and the depth of superficial nerve trunks. Results. The analysis confirmed the established SMAS types (I–V) in the cheek, parotid gland, and neck, confirming the validity of the method. Two distinct, sequentially arranged structures were identified on the forehead, proposed as new types. Type VI (neurovascular arborization) is a discrete fan-shaped structures with a central collagen core surrounding a neurovascular bundle, showing positive S100 staining. These structures, spaced approximately 2.2 mm apart, function as true retaining ligaments. Type VII (fibroseptal) SMAS patterns is vertically oriented, purely fibrous septa (retinacula cutis) connecting the aponeurosis to the dermis, devoid of neural elements, and spaced approximately 9.2 mm apart. Importantly, the superficial nerve trunks were located at an average depth of only 1.09 mm (range: 0.57–1.97 mm) from the skin surface. Conclusion. This study identified two novel SMAS patterns in the forehead—neurovascular arborization (type VI) and fibroseptal (type VII)—supporting the expanded functional seven-type classification of the SMAS. The extremely superficial location of the forehead nerves (average 1.1 mm) defines a critical “danger zone” for aesthetic procedures. These findings provide a refined anatomical basis for improving the precision and safety of both surgical and minimally invasive facial procedures.

## 1. Introduction

### 1.1. The Superficial Musculoaponeurotic System (Smas)

The Superficial Musculoaponeurotic System (SMAS) is of fundamental importance to contemporary plastic and reconstructive facial surgery. The SMAS was first described in 1976 by Mitz and Peyronie [1]. It is defined as an organized fibro-muscular network situated between the subcutaneous adipose tissue and the deeper facial tissues. This complex, three-dimensional structure consists of collagen and elastin fibers, muscle elements, and adipocytes. It provides support for the soft tissues of the face and, most importantly, acts as a distributor of tension by transmitting the contraction of facial muscles to the skin, thereby enabling complex facial movements [1].

Initial descriptions of the SMAS centered on the parotid and cheek regions, where a continuous fibro-muscular plate could be identified that could be surgically separated from the underlying parotid gland and masticatory muscle. This finding led to a transformation in rhytidectomy (facelift) procedures, moving away from the conventional approach of simple skin excision towards more substantial SMAS manipulations, such as plication, imbrication, and SMASectomy. This approach has been demonstrated to yield more natural and enduring rejuvenation outcomes due to its focus on the underlying cause of gravitational ptosis, namely the weakening of the facial structures that provide support [1].

### 1.2. Current Paradigms and Classifications of Smas

Since its initial description, the conceptual understanding of the SMAS has evolved from a uniform anatomical layer into a complex, heterogeneous system. Contemporary research indicates that the SMAS is not a monolithic structure; rather, it exhibits significant regional variability in both morphology and histology. This structural diversity reflects the specialized functional requirements of different facial zones, balancing the need for mobility with structural support.

To systematize these regional variations, several classification models have been proposed:

The Ghassemi Classification (2003): Ghassemi et al. identified two primary structural patterns based on their relationship to the nasolabial fold. Type I SMAS, located laterally, consists of vertically oriented fibrous septa enmeshing large adipocyte lobules. In contrast, Type II SMAS, found medially, is characterized by a dense, fiber-rich network of collagen and muscle elements with sparse fat content [2].

The Sandulescu Classification (2023): Expanding upon these findings using a Macaca mulatta model, Sandulescu proposed a more granular five-type classification. This model identifies specialized variants, including Type III (fat-free vertical septa in the eyelids), Type IV (parallel fibrous septa in the parotid region), and Type V (the cervical SMAS, where it integrates with the platysma) [3].

These paradigms underscore a fundamental biomechanical principle: the architecture of the SMAS is precisely adapted to local demands—facilitating gliding and volume displacement in the cheeks while ensuring firm, fine-tuned adhesion in the periorbital and perioral zones. Histologically, this regionalization is defined by specific gradients in elastin concentration, the ratio of Type I to Type III collagen, and the distribution of interstitial adipose tissue.

### 1.3. The Cranial Extension of the Smas: Anatomical Continuity vs. Homology

The anatomical continuity of the SMAS into the upper third of the face remains one of the most persistent subjects of terminological confusion in plastic surgery. While the anatomy of the midface SMAS is well-characterized, its relationship with the galeo-frontal complex is a point of contention.

A fundamental contradiction exists in the literature: many anatomical models describe the SMAS as a continuous, uninterrupted sheet that transitions cranially into the temporoparietal fascia (TPF) and the galea aponeurotica, effectively incorporating the frontalis muscle as its superior muscular component [1]. Conversely, other authoritative sources argue that the forehead and temple are not covered by the SMAS in the same histological sense as the parotid-cheek region, noting distinct embryological and structural differences [4].

This ambiguity arises from the transition of the SCALP layers into the specialized architecture of the temple and forehead. In the temporal region, the complexity increases significantly; surgeons must navigate multiple distinct planes, including the superficial and deep temporal fasciae and their intervening fat pads. A direct extrapolation of the “cheek SMAS” into this region is often inadequate [5]. This raises a critical question: is the “forehead SMAS” a true homologue (derived from the same embryological primordium) or a functional analogue (a separate structure performing a similar biomechanical role)? This lack of nomenclatural consensus necessitates a more granular, targeted histological investigation.

### 1.4. The Facial Retaining System

Concurrent with the development of the SMAS concept, the theory of the facial retaining system was established to explain the fixation of soft tissues to the underlying facial skeleton. This system is composed of two primary structural categories: true retaining ligaments and a diffuse network of fibrous septa known as the retinacula cutis [6].

True retaining ligaments are robust, fibrous bands that provide critical “anchor points” for the face. They are classified by their origin:

Osteocutaneous ligaments (e.g., zygomatic and mandibular) arise directly from the periosteum.

Fasciocutaneous ligaments (e.g., masseteric) originate from the deep fascia.

These structures penetrate perpendicularly through all facial layers, weaving into the dermis to limit gravitational displacement and divide the face into distinct anatomical spaces and fat compartments [6]. Microscopically, they exhibit a characteristic “tree-like” architecture: a dense periosteal “root” that bifurcates into a complex arborization of smaller fibers as it approaches the skin surface [6].

The retinacula cutis represents a more pervasive, though less dense, network of fibrous septa that connects the dermis to the underlying superficial fascia or mimetic muscles [7]. Recent research emphasizes that the retaining ligaments and the retinacula cutis are parts of a continuum; the ligaments represent localized reinforcements of this fibrous system situated at the boundaries of facial glideplanes [7]. Together, the SMAS and the retaining system function as a single biomechanical complex, where the ligaments provide the necessary stability and the SMAS facilitates the coordinated movement and expression of the facial soft tissues.

### 1.5. Rationale for the Study

Given the persistent anatomical and terminological ambiguity surrounding the SMAS in the forehead, as well as its complex relationship with the facial retaining system, there is a clear need to characterize the specific fibro-architectural structures connecting the dermis to the deep fascial layers in the upper third of the face.

The central research question of this study pertains to the fundamental nature of these structures: do they represent unique, previously undescribed morphological variants of the SMAS, or are they regional manifestations of the existing retaining system (e.g., retinacula cutis or localized retaining ligaments)? Clarifying this distinction is of paramount importance to both anatomical science and clinical facial surgery.

Utilizing body donors and histological analysis, this study aims to describe two potentially distinct morphological variants within the forehead:

A purely fibrous variant, characterized by dense connective tissue septa.

A complex, fan-shaped variant, which appears to serve as a conduit for its own neurovascular elements.

We propose that these structures should be integrated into an expanded, regionalized classification of the SMAS. Such an expansion would move beyond the original descriptions centered on the midface [1] to create a more comprehensive and functionally oriented anatomical map. The ultimate objective is twofold: to confirm the presence of these distinct SMAS-like layers in the upper face and to categorize this support system within a unified, functional paradigm of the superficial musculoaponeurotic system.

## 2. Materials and Methods

The study was conducted in accordance with the principles of the Declaration of Helsinki and received approval from the Local Ethics Committee [Sechenov University: protocol No. 149 dated 13 November 2023].

### 2.1. Sample Collection and Preparation

Facial tissue specimens (n = 30) were harvested ex vivo from 12 male and 18 female body donors (age range: 25–70 years). The mean age was 52.4 ± 13.8 years and the cohort is stratified into three clinically relevant age subgroups: 25–40 years (n = 7), 41–60 years (n = 12), and >60 years (n = 11).

To minimize post-mortem autolysis, all samples were obtained within 24 h of death. Specimens with a history of facial trauma, prior plastic surgery, or pathological processes that might distort the native anatomy were excluded. Full-thickness tissue blocks—incorporating skin, subcutaneous adipose tissue, SMAS, deep fascia, and muscle—were excised from five standardized regions: parotid, buccal, temporal, frontal, and cervical. Frontal samples were taken medial to the temporal crest, as the anatomy changes significantly at that transition point.

Cause of death: cardiovascular causes (n = 18, 60%), malignant neoplasms (n = 7, 23%), other (n = 5, 17%). None had conditions affecting facial soft tissues. Post-mortem interval: mean 12 ± 5 h (all within 24 h). BMI: systematic data not recorded. For donors with available data (n = 14), BMI ranged 21.3–29.7 kg/m^2^ (mean 25.1). Donors with obvious obesity or cachexia were excluded. Skin type: predominantly Caucasian/European descent (Fitzpatrick II–III).

**Figure 1:**
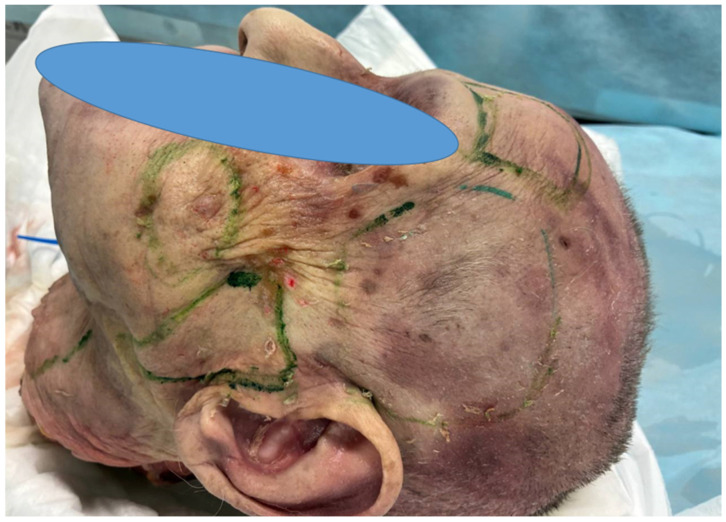
Marking the face before taking tissue samples for microscopic examination.

The areas of tissue sampling for microscopic examination were marked with a colored marker on the head of the corpse (Figure 1).

### 2.2. Histological Processing

Body donors were immediately fixed in 10% neutral buffered formalin for 24–48 h at room temperature. Following fixation, tissues underwent automated dehydration in a graded ethanol series (70% to 100%), clearing in xylene, and infiltration with paraffin wax. Serial sections (3 µm) were cut using a semi-automatic rotary microtome (NM 355 S, Thermo Scientific, Dreieich, Germany) and mounted on adhesive-coated slides (Gerhard Menzel GmbH, Braunschweig, Germany) to prevent tissue delamination during staining.

### 2.3. Histochemical Analysis

For general morphological assessment, sections were stained with Hematoxylin and Eosin (H&E) using a Gemini automatic stainer (Thermo Scientific, Basingstoke, UK). To achieve detailed visualization of the connective tissue architecture and muscular components, the following histochemical techniques were employed:

Van Gieson’s Stain: Utilized to differentiate collagen fibers (stained red/pink) from muscle tissue and cytoplasm (stained yellow-brown).

Masson’s Trichrome: Utilized for the high-contrast discrimination of collagen (blue/green), muscle fibers (red), and cell nuclei (black).

These staining modalities facilitated both qualitative assessment of the SMAS architecture and quantitative analysis of the fibrous-to-muscular ratios across the various facial regions.

### 2.4. Immunohistochemical Analysis (Ihc)

To identify and map peripheral nerve structures within the SMAS variants, immunohistochemical staining was performed using polyclonal antibodies against the S100 protein (a marker of Schwann cells and mature neuroglia). Representative specimens from each facial region (n = 6 per location) were selected for analysis.

The IHC procedure was conducted as follows:

Antigen Retrieval: Heat-induced epitope retrieval (HIER) was performed using a high-pH buffer (PRIME HIER TRIO, pH 9.0) in a PT module (Thermo Scientific, UK) at 98–99 °C for 20 min.

Staining Protocol: Reactions were carried out on an automated Autostainer (Thermo Scientific, UK) according to the manufacturer’s validated protocols.

Detection and Visualization: A universal two-step HRP/DAB detection system (PrimeVision, PrimeBioMed) was employed. 3,3′-Diaminobenzidine (DAB) served as the chromogen, producing a distinct brown precipitate at antigenic sites.

Counterstaining: Nuclei were counterstained with Mayer’s hematoxylin to provide morphological contrast. Specimens were then dehydrated, cleared in xylene, and mounted with a synthetic polystyrene medium.

Negative control: a serial section processed identically but with primary antibody replaced by non-immune rabbit serum—no DAB signal.

Positive control: sections of sciatic nerve included in each batch—intense S100 staining.

Criteria for S100 positivity: positive = clear brown cytoplasmic/nuclear staining in cells with Schwann cell morphology, intensity ≥ positive control; negative = no staining or faint diffuse background equal to negative control. Assessment was binary (positive/negative) because the research question was whether neural elements are present. Two investigators (D.K. and E.O.B.) achieved 100% concordance.

### 2.5. Microscopic and Morphometric Analysis

Histological slides were initially evaluated using an Axio Imager M2 light microscope (Carl Zeiss Microscopy, Jena, Germany). For high-resolution digital archiving and quantitative analysis, slides were scanned with a PANNORAMIC 250 Flash III digital scanner (3DHISTECH, Budapest, Hungary) at a native magnification of 20× (producing a 200× digital equivalent).

Morphometric analysis was conducted using PANNORAMIC Slide Viewer software (version 1.15.3). To characterize the fibro-vascular architecture of the forehead SMAS, the following parameters were measured:

Inter-septal distances (at the base and at mid-branching points).

The center-to-center distance between adjacent fibrous septa.

The depth of superficial nerve trunks relative to the stratum corneum.

The linear distance between adjacent nerve trunks.

Statistical Analysis: Quantitative data were processed to determine the arithmetic mean (M), standard deviation (SD), median (Me), and 95% confidence intervals (CI).

Measurement definitions:

− Inter-structural distance (Type VI): shortest linear distance between geometric centers of two adjacent Type VI bases at deep fascia level.
− Inter-septal distance (Type VII): measured at the base (midpoints at origin from galea) and at mid-branching zone.
− Nerve trunk depth: perpendicular distance from stratum corneum surface to center of the most superficial S100-positive fascicle.
− Inter-nerve distance: center-to-center horizontal distance between adjacent S100-positive nerve cross-sections.

Intra- and inter-rater consistency: measurements performed independently by two investigators (D.K. and P.S.) on two occasions ≥2 weeks apart. Intra-rater ICC: 0.94 (95% CI 0.89–0.97) for D.K., 0.92 (0.86–0.96) for P.S. Inter-rater ICC: 0.91 (0.84–0.95).

## 3. Results

**Figure 2:**
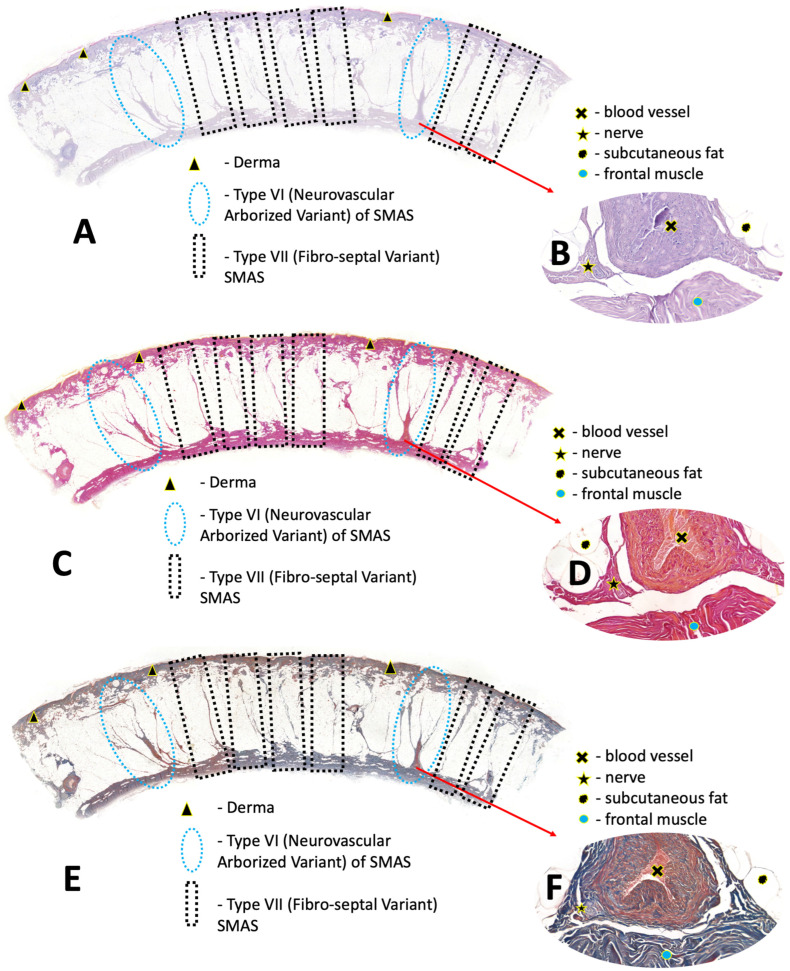
Histological images of the forehead skin demonstrating Type VI (Neurovascular Arborized Variant) and Type VII (Fibro-septal Variant) SMAS structures. (**A**,**C**,**E**) Low-magnification overview of serial sections from the same tissue block stained with H&E (**A**), Van Gieson (**C**), and Masson’s trichrome (**E**), respectively. The fan-shaped arborized structures with a broad base (Type VI) are visible alongside thinner, vertically oriented fibrous septa (Type VII). Van Gieson staining highlights collagen fibers in red; Masson’s trichrome differentiates collagen (blue) from muscle fibers (red). (**B**,**D**,**F**) Higher magnification of the base of the central rod within a Type VI structure from the same serial sections, stained with H&E (**B**), Van Gieson (**D**), and Masson’s trichrome (**F**), respectively. The central rod incorporates vascular channels comprising arterioles and/or venules with well-defined walls and lumens containing erythrocytes, alongside a nerve trunk. Van Gieson staining (**D**) demonstrates the dense collagenous composition of the rod (red), while Masson’s trichrome (**F**) reveals the spatial relationship between collagen (blue) and muscle elements (red) at the rod’s periphery.

Histological and immunohistochemical examination of tissue samples from various anatomical areas of the face confirmed existing ideas about the regional heterogeneity of SMAS and revealed two previously undescribed morphological structures in the forehead area that do not fit into the existing Type I–V classifications. These structures are proposed to be classified as new types of SMAS (Figure 2).

### 3.1. General Histotopography of the Smas in Established Areas

Analysis of specimens from the cheek, parotid, and cervical regions yielded results consistent with established literature, validating the study’s methodology.

Cheek (Lateral to the NLF): All samples exhibited Type I SMAS (Ghassemi/Sandulescu). This consisted of vertically oriented fibrous septa originating from the fascia masseterica and weaving into the dermis, compartmentalizing the subcutaneous fat into voluminous lobules.

Parotid Region: A Type IV structure was identified, characterized by dense fibrous septa running parallel to the skin surface with firm adhesion to the fascia parotidea.

Cervical Region: A Type V morphology was observed; the SMAS was inextricably integrated with the platysma, forming parallel plates connected by vertical bridges that transmit muscular tension to the skin.

### 3.2. Specific Histological Findings in the Forehead Region

Unlike the lower face, the forehead tissues revealed two distinct, sequentially occurring supporting structures. We propose these be categorized as region-specific SMAS variants.

#### 3.2.1. Type Vi: Neurovascular Arborized Variant

**This variant is a functionally complex, organized structure that serves as a protective conduit for neurovascular elements.**

1. Morphology: These structures are discrete, macroscopically visible formations. Each structure has a single broad and sturdy base that is attached to the superficial frontal fascia. From this base, the structure diverges in a fan-like (arborized) pattern into 3–5 thinner processes, which also penetrate the hypodermis and intertwine with the reticular layer of the dermis.

2. Histologically, a central rod is clearly identifiable in the center of the broad base and in the proximal parts of the processes. This rod contains vascular channels at its base—arterioles and/or venules with clearly distinguishable walls and lumens filled with erythrocytes, as well as a nerve trunk. Dense collagen fibers radiate from this central rod, forming processes. This morphology fully corresponds to the description of true retaining ligaments, which serve as conduits for perforating vessels. IHC analysis showed intense positive staining for S100 in the nerve trunk at the base of the central rod. This convincingly proves the presence of an integrated nerve component accompanying the vessels. Thus, these structures represent full-fledged neurovascular bundles enclosed in a fibrous sheath.

3. Morphometry (n = 23): The mean distance between these structures was 2230 ± 425 μm (range: 1500–2975 μm).

#### 3.2.2. Type Vii: Fibro-Septal Variant

This variant is characterized by a system of robust, purely fibrous septa.

1. Morphology: These are vertically oriented connective tissue bands that connect the fascia of the frontal muscle to the dermis. They originate from the supra-cranial aponeurosis (galea aponeurotica) and the superficial frontal fascia, which form the bed of the frontal muscle. They penetrate the subcutaneous adipose tissue and are woven directly into the reticular layer of the dermis.

2. Histological examination reveals that these septa are composed of dense, compact bundles of collagen fibers. Staining with Van Gieson’s stain results in a bright red appearance, while Masson’s stain yields a blue/green hue. The structures under scrutiny are characterized by an absence of cellular components, with a notable absence of integrated muscle fibers. These structures are found to contain a limited number of fibroblasts. The process of division of the subcutaneous fat layer into smaller compartments is an effective method of achieving this. Morphologically, these structures fully correspond to the classical description of retinacula cutis. When stained for S100 protein, these fibrous septa were consistently negative. This finding serves to confirm the absence of significant integrated nerve fibers in their composition, thus distinguishing them from the second variant described. Morphologically, these structures represent a specialized form of retinacula cutis.

3. Morphometry (n = 5): The mean inter-septal distance at the base was 9166 ± 543 μm, with a range of 8283–9779 μm. The mean average distance in the branching zone (middle) was found to be 9951 ± 723 μm and this confirms a fan-shaped architecture as they approach the dermis.

For a clear comparison, the key characteristics of the two newly described variants are summarized in Table 1.

#### 3.2.3. Spatial Distribution of Types Vi and Vii

Type VI structures were found predominantly in the lower and central forehead, concentrated in a band from the supraorbital ridge to approximately 2–3 cm above it. Density was highest in the paramedian zone (1–3 cm lateral to midline), corresponding to emergence points of supraorbital and supratrochlear neurovascular bundles. Laterally, density decreased approaching the temporal crest. In the upper forehead (above the mid-frontal line), Type VI was sparse or absent. Type VII structures were distributed more diffusely across the entire forehead, from supraorbital margin to frontal hairline, with relatively uniform spacing. No significant medial-to-lateral gradient was observed. Type VII septa were particularly prominent in the midline glabellar region. Anatomical landmarks used: supraorbital ridge, supraorbital notch/foramen, temporal crest (linea temporalis), frontal hairline, midline (glabellar midpoint).

**Figure 3:**
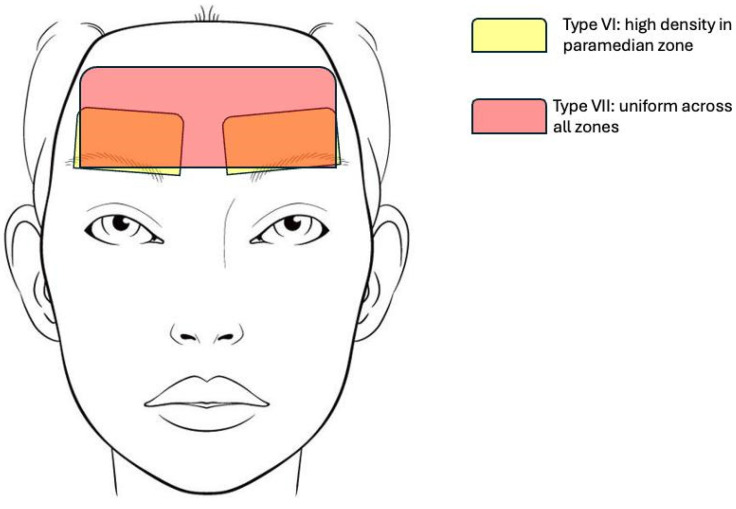
Topographic distribution map of Type VI (Neurovascular Arborized, yellow area) and Type VII (Fibro-septal, red area) SMAS variants across the forehead region.

A topographic distribution map is provided in Figure 3.

### 3.3. Topography of the Superficial Nerve Network

The analysis of nerve trunk depth yielded critical data for clinical practice. Contrary to the commonly accepted view in classical anatomy, the nerves are located much more superficially (Table 2).

The statistical data set comprised 30 measurements.

Average depth from the skin surface: The mean value was found to be 1092 ± 272 μm (approximately 1.1 mm).

The minimum recorded depth was determined to be 572 µm (0.57 mm).

The maximum depth of the structure is 1970 µm (1.97 mm).

Interpretation: In all cases, the nerve trunks were located within 2 mm of the skin surface, and in some cases at a depth of only 0.5–0.6 mm, which corresponds to the level of the deep dermis or the border between the dermis and hypodermis.

**Figure 4:**
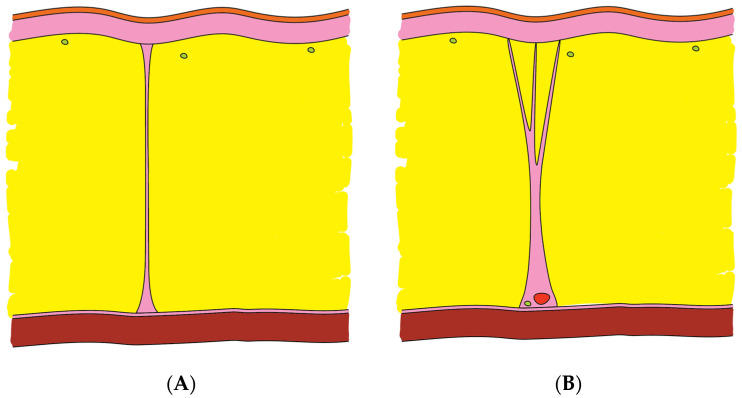
Schematic representation of two newly described variants of the Superficial Musculoaponeurotic System (SMAS). (**A**) Type VI: Neurovascular Arborized Variant. This structure features a single broad and robust base anchored to the superficial frontal fascia. From this base, it diverges in a fan-like (arborized) pattern into 3–5 thinner processes that penetrate the hypodermis and interdigitate with the reticular layer of the dermis. A central rod is distinctly visible within the broad base and the proximal segments of the processes; this rod incorporates vascular channels at its base, comprising arterioles and/or venules with well-defined walls and lumens containing erythrocytes, alongside a nerve trunk. (**B**) Type VII: Fibro-septal Variant. This structure consists of vertically oriented connective tissue septa that link the fascia of the frontalis muscle to the dermis. These septa are devoid of substantial cellular components, notably lacking integrated muscle fibers, and contain only a limited population of fibroblasts. They function to partition the subcutaneous adipose layer into discrete compartments.

For a clear comparison, the key characteristics of the two newly described variants are summarized in Table 2 and at Figure 4.

## 4. Discussion

This study corroborates the paradigm of regional SMAS heterogeneity and identifies two distinct supporting structures in the forehead that warrant classification as novel SMAS variants. Our findings align with the hypothesis proposed by Minelli et al. (2024), suggesting the SMAS is not a strictly independent layer but a specialized functional modification of the superficial fascia and retinacula cutis [7].

Type VI represents a distinctive neurovascular penetrating ligament. Its identification provides a histological rationale for the observation by Knize [8] that forehead “lines of adhesion” frequently coincide with sensory nerve exit points. Consequently, the surgical release of these ligaments to achieve tissue mobilization inherently risks the transection of terminal nerve branches, as evidenced by our S100+ immunohistochemical data.

Type VII appears to be a specialized, hypertrophied variant of the retinacula cutis. This structure is biomechanically optimized for the rigid fixation of forehead skin to the m. frontalis. This high-fidelity attachment prevents the skin from “gliding” independently of the muscle, ensuring that muscular contraction is directly translated into facial expression. This system is further supported by the concept of a Deep Musculoaponeurotic System (DMAS) [9,10], suggesting a continuous functional unit extending from the dermis through the muscle to the periosteum.

**Clinical Implications: The “1.1 mm Danger Zone”**

Our morphometric data reveals that the mean depth of these superficial nerve trunks is a mere 1.1 mm (1092 µm), with a minimum recorded depth of 0.6 mm (572 µm). This finding has critical safety implications for non-surgical aesthetics:

Ablative Laser Resurfacing: Traditional CO2 or Erbium lasers operating at depths of 600–800 µm (targeting the reticular dermis) place patients—particularly those with thin skin—at direct risk for thermal denervation of Type VI structures.

Microneedling Radiofrequency (MNRF): Standard settings of 1.5–2.0 mm in the forehead region are highly likely to cause direct coagulative necrosis of these nerve trunks. Considering this, we suggest that depth settings exceeding 1.0 mm in the lower forehead are potentially unsafe.

Intradermal Injections: For techniques like “blushing” or mesotherapy, injections should be restricted to a depth of <1 mm to avoid the neurovascular passage zone.

### 4.1. Contextualization of Findings Within the Layer-by-Layer Anatomy of the Forehead

The layered anatomy of the forehead is uniquely complex, representing a structural transition from the five-layered SCALP architecture to the multi-planar specialized systems of the midface [11]. It is imperative that our findings are interpreted within this hierarchical framework:

Type VI (Neurovascular Arborized): These function as primary structural anchors. Originating from the periosteum (Layer 5) and traversing all overlying layers to the dermis, they provide deep fixation of the entire soft-tissue complex to the frontal bone. Their robust architecture serves as a protective conduit for neurovascular bundles, ensuring skeletal stability while safeguarding vital structures.

Type VII (Fibro-septal): These represent functional musculocutaneous tethers. These vertical elements connect the dermis (Layer 1) to the galea aponeurotica and frontalis muscle (Layer 3). This close skin-to-muscle coupling provides the superficial adhesion necessary to translate muscular contraction into the formation of horizontal forehead rhytids.

This distinction is clinically significant: Type VI establishes the boundary for deep tissue compartments and the spread of subgaleal fluid, while Type VII defines the “danger zone” for superficial aesthetic interventions, such as laser depth and the precision of intradermal injections.

### 4.2. The Question of Novelty: Synthesis with Existing Concepts

A critical evaluation is required to determine whether the structures described herein are truly “novel” or represent refined characterizations of known anatomical elements.

#### 4.2.1. The Neurovascular Arborized Variant (Type Vi) as a True Retaining Ligament

Type VI exhibits striking structural parallels to established true retaining ligaments, such as the zygomatic and orbital retaining ligaments [6]. Key shared features include:

Origin: Attachment to the deep fascia or periosteum.

Trajectory: A perpendicular path relative to the skin surface.

Arborization: Fan-like branching into the reticular dermis [6].

Neurovascular Relationship: Acting as a protective conduit for vital structures.

We propose that Type VI is a specific morphological designation for the true retaining ligaments of the frontal region, previously described inconsistently as “supraorbital ligamentous adhesions” [6]. These ligaments serve as specialized “gateways” for the perforating branches of the supraorbital and supratrochlear arteries, veins, and nerves as they emerge from the submuscular plane to supply the forehead and scalp [12]. The novelty of our study lies in the detailed histological characterization of these structures and the proposal for their conceptual integration into a regionalized SMAS classification.

#### 4.2.2. The Fibro-Septal Variant (Type Vii) as Specialized Retinacula Cutis

Structurally, Type VII is homologous to the retinacula cutis—the ubiquitous network of fibrous septa connecting the dermis to the deep fascia [7,13]. However, we argue that in the forehead, this system achieves a level of organization and functional density that justifies its inclusion in the SMAS paradigm. The specific vertical orientation of these septa ensures the high-fidelity coupling between the skin and the m. frontalis, serving as the anatomical substrate for persistent transverse rhytids [14]. Furthermore, these elements comprise the dense connective tissue found within the midline bifurcation of the frontal muscle bellies observed in most individuals [15].

### 4.3. Arguments for an Expanded, Functional Smas Classification

We contend that the definition of the SMAS should be functional rather than strictly morphological. If the SMAS is defined as the system responsible for soft-tissue support and the biomechanical transmission of muscular force, then Types VI and VII are its primary components in the upper third of the face. Excluding them based on histological differences from the “classic” cheek SMAS ignores the principle of regional specialization.

This approach merges the two dominant theories of facial support—the SMAS theory and the Retaining Ligament theory—into a unified model. In this framework, the SMAS is an integrated network of anchors (True Ligaments; Type VI), slings (Fibromuscular plates; Types I–IV), and tethers (Dense Septa; Type VII). Based on this holistic understanding of facial biomechanics, we propose expanding the existing five-type classification to a seven-type model (Table 3).

We propose a functional definition: any structure should be classified as SMAS if it simultaneously (a) originates from deep fascia or periosteum; (b) inserts into the dermis; and (c) participates in biomechanical transmission of muscular force or soft-tissue fixation. Under this definition, Type VI and Type VII are SMAS components.

### 4.4. Clinical and Functional Significance

The proposed seven-type classification offers a localized framework for improving safety and predictability in facial rejuvenation.

1. Pathomechanics of Forehead Aging With senescence, the structural integrity of both the true ligaments (Type VI) and the dense fibrous septa (Type VII) diminishes. The weakening of these attachments facilitates gravitational brow ptosis and the deepening of glabellar lines. As the Type VII fibro-septal anchors lose their tensile strength, the skin’s firm attachment to the m. frontalis is compromised, contributing to the transition from dynamic to static rhytids.

2. Significance for Surgical Procedures

Mobilization and Lift: Effective tissue elevation in endoscopic or open brow lifts requires the precise identification and release of the Type VI true retaining ligaments (the supraorbital ligamentous adhesions).

Dissection Planes: Understanding the architecture of these variants assists the surgeon in selecting the optimal plane—subcutaneous, subfascial, or subperiosteal—to ensure adequate mobilization while protecting the neurovascular “palisades” formed by Type VI structures.

Nerve Preservation: The Type VI variant serves as a critical anatomical landmark. Its medial proximity to the supraorbital and supratrochlear bundles [12] and its lateral relationship to the frontal branch of the facial nerve [14] make it an essential “sentinel” structure for avoiding iatrogenic injury.

3. Significance for Minimally Invasive Procedures

Neuromodulators (BoNT): The fibers of the m. frontalis are closely integrated with Type VII septa. The injection depth (superficial vs. intramuscular) dictates whether the toxin affects the muscle fibers or the force-transmitting septa, potentially explaining variations in clinical efficacy and “brow freeze” vs. natural expression.

Injectable Fillers: Type VI structures represent “no-go zones” for deep bolus injections due to the risk of compressing or cannulating the encased supraorbital and supratrochlear vessels. This provides a clear anatomical rationale for avoiding catastrophic complications such as vascular occlusion or blindness [18]. Conversely, superficial filler placement can soften wrinkles caused by Type VII septal tethering.

### 4.5. Biomechanics and Clinical Interpretation

The Type VI variant acts as a high-modulus “strain shield,” utilizing its dense collagenous rod to protect encapsulated neurovascular bundles from mechanical shear and compression during facial animation. Its arborized canopy facilitates even tension distribution, translating muscular force into synchronized brow movement without localized skin dimpling. Collectively, this high-density palisade provides significant structural rigidity, explaining both the limited tissue glide and the resistance to blunt dissection in the lower forehead. Clinical Interpretation: the high frequency of occurrence (~every 2 mm) makes it impossible to perform “blind” separation of tissues in the subcutaneous layer without damaging these structures. The dense Type VI palisade explains the difficulty of blunt dissection in the lower third of the forehead.

Structural configuration of Type VII facilitates uninterrupted transmission of m. frontalis contraction to the dermis, thereby establishing the anatomical foundation for the development of horizontal forehead wrinkles. Clinical Interpretation: large Type VII fibrous “anchors” are located at intervals of approximately 9–10 mm. This phenomenon corresponds to the macroscopic spacing of deep horizontal furrows on the forehead. The widening of the distance in the middle (9.9 mm versus 9.1 mm at the base) indicates a fan-shaped divergence of the septa towards the skin surface and a high risk of superficial bleeding or denervation.

### 4.6. Limitations and Future Directions

This study has several limitations. First, the sample size was relatively small and consisted primarily of elderly donors, which may reflect age-related architectural thinning. Second, as a static cadaveric model, it does not account for in vivo dynamic tissue compliance. Due to small subgroup sizes (e.g., n = 7 in the youngest group), formal inferential testing was not performed to avoid type II error. Data are presented descriptively.

While H&E and Masson’s Trichrome confirmed the presence of vascular channels in Type VI, future research should employ endothelial-specific markers (e.g., CD31) to further map the microvasculature. Future studies should also utilize ultra-high-frequency ultrasound (U-SMAS) to visualize these palisades in vivo [19,20,21]. Finally, correlating the precise density of Type VII septa with individual wrinkle patterns could facilitate the development of personalized, “topographically-guided” rejuvenation protocols.

Due to small subgroup sizes (e.g., n = 7 in the youngest group), formal inferential testing was not performed to avoid type II error. Likewise, the sample did not allow reliable gender-stratified analysis; descriptive assessment revealed no obvious gender-related morphological differences. Data are presented descriptively.

In Table 4 we systematize and summarize the information on SMAS.

## 5. Conclusions

This study provides a detailed anatomical and histological characterization of two distinct supporting structures in the human forehead, proposed as novel additions to the SMAS classification: Type VI (Neurovascular Arborized) and Type VII (Fibro-septal). Type VI represents true retaining ligaments that provide deep skeletal fixation of soft tissues and serve as vital protective conduits for neurovascular bundles. Conversely, Type VII corresponds to a highly organized network of specialized retinacula cutis responsible for the high-fidelity adhesion of the skin to the m. frontalis.

The central conclusion of this work is that the SMAS should be re-envisioned as a comprehensive, functional system rather than a regionalized anatomical layer. By incorporating these specialized retaining structures of the upper third of the face, this model unifies previously disparate theories of facial support into a single, coherent paradigm. This expanded understanding provides a more precise and clinically relevant anatomical map, enhancing the safety and efficacy of both surgical and minimally invasive facial rejuvenation procedures.

## Figures and Tables

**Table 1:** Characteristics of the proposed SMAS types.

Characteristic	Proposed Type VI (Neurovascular Arborized)	Proposed Type VII (Fibroseptal)
**Origin**	Deep fascia/Periosteum	Deep fascia (Galea aponeurotica/Fascia temporalis)
**Weaving**	Derma	Derma
**Macromorphology**	Strong, discrete, fan-shaped (arborized) structures	Thin, linear, diffuse septa
**Histological composition**	Fibrous adipose tissue with a central core	Dense collagen, acellular
**Vascular part (H&E)**	Integrated, centrally located vessels	Missing or random
**Neural part (IHC, S100)**	Positive (in the central core)	Negative
**Functional analogue**	True retaining ligament	Retinacula cutis

**Table 2:** The following is a synopsis of morphometric indicators of forehead structures.

Parameter	M (μm)	SD (μm)	Min (μm)	Max (μm)
**Type VI (base-to-base distance)**	2170	425	1500	2975
**Type VII (mid-to-mid distance)**	9166	543	8283	9779
**Depth of the nerve trunk (from the surface of the epidermis)**	1092	272	572	1970
**Distance between nerve trunks**	1960	515	1028	2770

**Table 3:** Proposed extended classification of the human facial SMAS.

Type	Anatomical Location	Key Structural Characteristics	Main Function	References
**Type I**	Lateral border of the buccal region, parotid region	Vertical fibrous septa in adipose tissue	Providing mobility and volume	[16]
**Type II**	Lips, repioral region	Dense collagen-muscle network	Fine motor control, facial expressions	[16]
**Type III**	Eyelids	A thin, fat-free, loose fibroelastic network	Delicate skin adhesion	[16]
**Type IV**	Parotid and temporal regions	Fibrous septa parallel to the skin	Fixed anchorage	[17]
**Type V**	Neck	Parallel septa with vertical bridges, integrated by platysma	Transfer of platysma contractions	[18]
**Type VI (suggestion)**	lower/central forehead (paramedian zone)	Strong, arborized ligaments with a neurovascular core	Deep soft tissue fixation, neurovascular bundle guide	Current research
**Type VII (suggestion)**	entire forehead (diffuse, uniform spacing)	Thin, vertical fibrous septa (retinaculacutis)	Skin adhesion to muscle, wrinkle formation	Current research

**Table 4:** Systematic comparison of all seven SMAS types by composition, architecture, neural and vascular content, function, and clinical relevance.

SMAS Type	Anatomical Location	Tissue Composition	Architectural Pattern	Neural Content (S100)	Vascular Content	Functional Role	Primary Clinical Relevance
**Type I**	Lateral cheek, parotid region	Fibrous septa enclosing large adipocyte lobules	Vertically oriented fibrous septa	Absent (negative)	Small vessels within septa	Provides mobility and volume; allows gliding of subcutaneous fat	Safe plane for dissection in facelift; risk of contour irregularity if over-resected
**Type II**	Lips, perioral region	Dense collagen–muscle network (mixed fibromuscular)	Irregular, dense network with muscle fibers	Present (muscle-associated nerves)	Rich capillary network	Fine motor control; precise facial expressions (e.g., orbicularis oris function)	Avoid aggressive resection to prevent oral incompetence; neuromodulator injection guidance
**Type III**	Eyelids	Thin, fat-free fibroelastic tissue	Loose, delicate network	Sparse nerve endings	Minimal	Delicate skin adhesion; allows rapid, fine movements	Danger zone for filler injection; thin skin predisposes to visible lumps and Tyndall effect
**Type IV**	Parotid and temporal regions	Dense fibrous septa running parallel to skin	Parallel, sheet-like layers	Absent	Moderate	Fixed anchorage; limits skin mobility over parotid	Landmark for deep plane facelift; protects facial nerve branches
**Type V**	Neck	Parallel septa with vertical bridges, integrated with platysma	Vertical and horizontal bridging fibers	Present (platysma motor nerves)	Moderate	Transmits platysma contraction to skin; neck contouring	Essential for neck lift and platysmaplasty; risk of marginal mandibular nerve injury
**Type VI (proposed)**	Lower and central forehead (paramedian zone)	Dense collagen core with central neurovascular bundle	Fan-shaped (arborized)—single broad base diverging into 3–5 thinner processes	**Positive** (S100+ nerve trunk in central rod)	**Present** (arterioles/venules in central core)	Deep soft-tissue fixation; true retaining ligament; protects neurovascular bundles	**“1.1 mm danger zone”**—critical for laser, RF, microneedling, filler injection; release required for brow lift
**Type VII (proposed)**	Entire forehead (diffuse, uniform spacing)	Dense, compact collagen bundles; acellular, no muscle	Vertically oriented fibrous septa (retinacula cutis)	**Negative** (S100−)	Minimal (random, not integrated)	High-fidelity skin-to-frontalis coupling; transmits muscle contraction to skin; forms horizontal rhytids	Anatomical basis for forehead wrinkles; superficial injection plane (<1 mm) to avoid neurovascular structures; guides BoNT depth

## Data Availability

Data will be made available upon reasonable request by the corresponding author. Data is not publicly available due to privacy or ethical restrictions associated with anatomical material.
